# Dynamic Bandwidth Slicing in Passive Optical Networks to Empower Federated Learning

**DOI:** 10.3390/s24155000

**Published:** 2024-08-02

**Authors:** Alaelddin F. Y. Mohammed, Joohyung Lee, Sangdon Park

**Affiliations:** 1 Information Technology, Department of International Studies, Dongshin University, 67, Dongshindae-gil, Naju-si 58245, Republic of Korea; alaelddin@dsu.ac.kr; 2 Department of Computing, Gachon University, Seongnam-si 13120, Republic of Korea; 3 School of Electrical Engineering, Korea Advanced Institute of Science & Technology (KAIST), Daejeon 34141, Republic of Korea

**Keywords:** federated learning, PON, DBA, bandwidth management, 6G

## Abstract

Federated Learning (FL) is a decentralized machine learning method in which individual devices compute local models based on their data. In FL, devices periodically share newly trained updates with the central server, rather than submitting their raw data. The key characteristics of FL, including on-device training and aggregation, make it interesting for many communication domains. Moreover, the potential of new systems facilitating FL in sixth generation (6G) enabled Passive Optical Networks (PON), presents a promising opportunity for integration within this domain. This article focuses on the interaction between FL and PON, exploring approaches for effective bandwidth management, particularly in addressing the complexity introduced by FL traffic. In the PON standard, advanced bandwidth management is proposed by allocating multiple upstream grants utilizing the Dynamic Bandwidth Allocation (DBA) algorithm to be allocated for an Optical Network Unit (ONU). However, there is a lack of research on studying the utilization of multiple grant allocation. In this paper, we address this limitation by introducing a novel DBA approach that efficiently allocates PON bandwidth for FL traffic generation and demonstrates how multiple grants can benefit from the enhanced capacity of implementing PON in carrying out FL flows. Simulations conducted in this study show that the proposed solution outperforms state-of-the-art solutions in several network performance metrics, particularly in reducing upstream delay. This improvement holds great promise for enabling real-time data-intensive services that will be key components of 6G environments. Furthermore, our discussion outlines the potential for the integration of FL and PON as an operational reality capable of supporting 6G networking.

## 1. Introduction

Human life and industry in recent decades has experienced great advantages due to the development of communication systems and services. There exist several examples of these real-time technologies and services introduced in recent years, such as Smart Home Systems, Virtual Reality (VR), and Multiplayer Online Games. Hence, the communication networks need extra improvements to adopt these technologies. The fifth generation (5G) communication network deployed extensively; meanwhile, research and industry are researching the 6G communication network as a mega-network that is heterogeneous. Machine Learning (ML), service interaction, and performance will be increased in the case of 6G. Consequently, the integration of Artificial Intelligence (AI) and ML into 6G infrastructure can be an enormous value in communication networks [1]. As the networks mature to become more flexible, intelligent, and autonomous, they are also ready for advanced AI capabilities [2]. In this more connected future, the intertwining of AI with 6G technology is revolutionizing the vision of communication networks as well as services. It is predicted that such convergence would render revolutionary changes in interaction with technology (e.g., the comprehensive establishment of network intelligence, flexible distribution resources, and independent decision-making) [3]. Federated Learning (FL) is a distributed ML technique that enforces data privacy and enables training models to be learned collaboratively across various decentralized devices such as smartphones, edge devices, and IoT. Training in FL is performed on devices locally and independently using data that are stored therein. However, updates on a global model are aggregated via FL an aggregator or server which constructs the updated version of it back to devices for enhancing their local models. The global model continues to be updated via filtered local updates without any compromise in data privacy.

Optical Access Network will be evolved as part of communication network evolution to fulfill the required Quality of Service (QoS) with their necessary bandwidth demand for customers and industries [4]. Passive Optical Networks (PON) (e.g., Ethernet-PON (E-PON), Gigabit-PON (G-PON)) are connected through point-to-multi-point topology where an Optical Line Terminal (OLT) plays as a master device located at the central office and connected with many Optical Network Unit (ONU) through a passive optical splitter. In 6G, the network environment is heterogeneous, and it should uphold the rapid development of the number of connections and hungry bandwidth-demanding applications, which can increase the delay and bypass the delay requirement for delay-sensitive applications. PONs can play a significant role in 6G networks to overcome the rapid demand for high-speed and scalable communication networks. PON can support fronthaul and backhaul by providing cost-effective solutions for 5G and beyond [5]. The main purpose of the Dynamic Bandwidth Allocation (DBA) algorithm in PONs is to provide that each ONU grants its demanded bandwidth as much as possible more efficiently with a fair bandwidth distribution between connected ONUs. The interaction between the OLT and ONU is achieved by the Report control message from the ONU to report its demand, and a Gate control message from the OLT to grant an upstream bandwidth to ONUs. Therefore, the DBA should effectively manage the allocation of available bandwidth resources in real time according to ONUs’ demands while maintaining fairness and QoS. However, while a new type of traffic emerges (e.g., FL traffic), PON’s resource allocation and delay require further development to support 6G. In this direction, only a few research papers exist related to PON’s bandwidth utilization for FL that help illustrate DBA’s advantages, which describe how bandwidth is allocated in a PON network. For instance, the authors of [6] proposed PON’s bandwidth slicing to support FL. Based on FL training parameters, the proposed mechanism allocates the bandwidth to the ONUs in ascending order. However, for multiple FL tasks, more bandwidth slices are required. The authors of [7] introduced a Wavelength and Bandwidth Allocation (DWBA) algorithm for FL application support over EPON. The solution prioritizes FL traffic statically. The question raised from that research is how the DBA differentiates between FL and normal traffic demands and how the ONU manages that traffic (e.g., queuing and reporting).

In the PON standard, the design of the Gate control message is of significant importance. As outlined in the XGS-PON standard [8], the actual number of physical or logical queues implemented at the ONU to manage these data can vary and depend on the ONU’s internal architecture, the network’s quality of service requirements, and the entity receiving upstream bandwidth allocation, denoted as an allocation ID (Alloc-ID). However, from the OLT’s perspective for bandwidth allocation and management, each Alloc-ID represents one logical queue or buffer. This approach allows the OLT to treat all Alloc-IDs as independent entities at the same level of hierarchy for the purposes of upstream bandwidth assignment, providing a fair and organized distribution of bandwidth among all connected ONUs. Because the ONU is assumed to have multiple upstream queues, this can allow the DBA to provide different upstream bandwidths (grants) for an ONU. However, there have been limited research articles that explain how a DBA can utilize more grants for an ONU during a DBA cycle. For instance, the authors of [6] addressed this topic, but they did not comprehensively show this feature. Our article aims to address this gap. Thus, by efficiently using more than one grant during a DBA cycle, we can improve upstream bandwidth in PON for FL traffic.

The motivation for this research comes from the FL integration within 6G-enabled PON. FL allows collaborative training models without transferring raw data, which can provide privacy in 6G networks. The unique characteristics of PON, such as DBA, present a promising platform for implementing FL efficiently. This integration between FL and PON is expected to address the rapid bandwidth demands and improve delay performance, required in supporting real-time and bandwidth intensive applications in 6G. The contributions of this article are summarized as follows:

- We introduce the importance of PON in 6G. We also summarize existing work on FL under PON and recent DBA algorithms to support FL.
- Utilizing this analysis as a foundation, the incoming FL model update traffic is modeled to be aggregated at the ONU, and then the results are transferred to a specified upstream queue of the ONU.
- We introduce a novel PON DBA considering different queue management at the ONUs and send a Gate control message from the OLT with multi-grants for those queues.
- We highlight different open challenges for future research and wrap up the article.

The remainder of the paper is organized as follows. In Section 2, we present related work regarding DBA implementation and FL approaches in PON. Section 3 introduces a case study of our proposed DBA-based FL aggregation over PON and its simulation results. Section 4 presents open challenges of FL under PON, and we conclude our paper in Section 5.

## 2. Overview of PON-Empowered Federated Learning

A few existing solutions have been implemented to deal with FL for optimal bandwidth utilization in PON. This article classifies the research methods into DBA optimization and FL aggregation.

### 2.1. Overall Process of PON-Empowered FL

**Figure 1:**
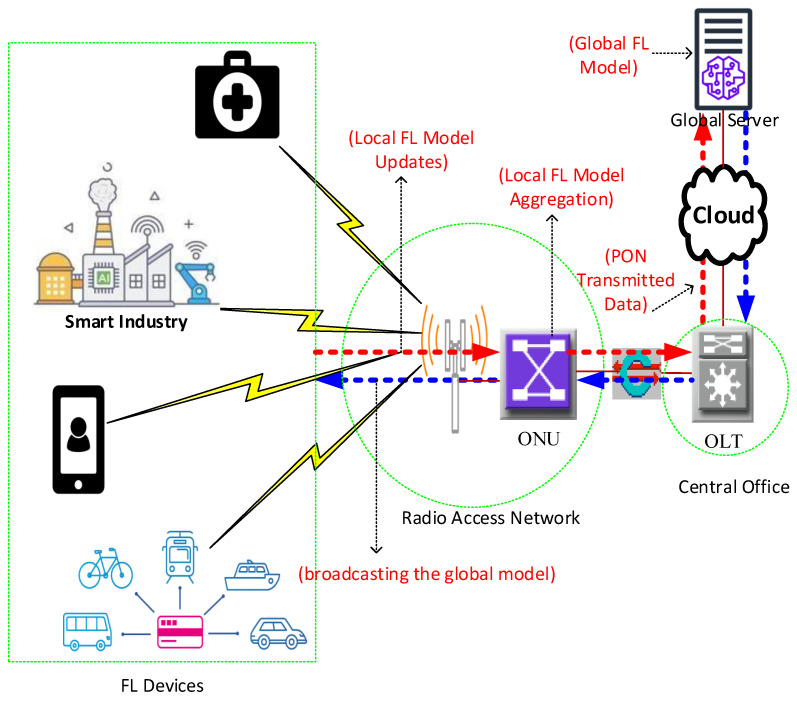
FL integration over PON in 6G.

Figure 1 shows glimpses of the role of PON in 6G. The figure illustrates a 6G integrated FL hierarchy across a PON. User devices (e.g., smartphones to IoT gadgets) train basic FL models and send updates to the ONUs at 6G base stations. The ONUs then aggregate local model updates and send them to the OLT, which aggregates FL models to the global server. The server refines the collective model and broadcasts the updated version through the OLTs and ONUs to user devices. An extensive survey in [9] presents the background of FL in networking, experimental simulations, and case studies. It also identifies challenges and future research directions in privacy, preserving FL in intelligent networking.

### 2.2. FL Aggregation over PON

FL aggregation’s primary objective revolves around developing methodologies that enable the seamless integration and processing of heterogeneous data sources while circumventing the need for centralized data consolidation.

A quintessential example in the work by [10] unveiled an edge-based multi-layer Hierarchical FL architecture that enables the execution of the conventional FL with model aggregation at several tiers. The suggested method is tested through several simulations, and the ultimate accuracy and loss of the models produced were contrasted with those of the conventional FL method. As a result, model aggregations may be carried out even when a set of edge nodes is not constantly linked to the cloud. Additionally, this solution enables model aggregations to communicate with the cloud less frequently, saving communication energy.

One more FL is developed based on the bandwidth-constrained client selection and scheduling presented in [11]. A two-step aggregation solution is introduced in which the local model parameters are first aggregated ONUs to enable scalable FL (SFL) over PONs, and then further aggregated by the central server at the central office. By doing this, the upstream bandwidth needed to transmit model parameters remains constant despite the number of IoT devices, resulting in high learning accuracy. The aggregated local models conveyed in PON make it difficult to read the unique local model for each client; hence, the suggested SFL may also enhance privacy.

### 2.3. DBA Optimization

In XGS-PON [8] standards, two kinds of DBA are mentioned: Status Reporting (SR) DBA and Traffic Monitoring (TM) DBA. The SR-DBA relies on the ONU’s buffer occupancy reports, whereas the TM-DBA relies on the traffic monitoring of an ONU. However, the implementation of the DBA, as outlined in [8], is subject to the discretion of individual OLT vendors. Thus, the OLT vendors can develop and utilize their own efficient DBA algorithms to meet diverse network environments and performance requirements. Thereby, vendors can introduce unique DBA and enhance network efficiency and service quality for customers. Nonetheless, this can also cause substantial variation in DBA performance and effectiveness across various OLT products due to the distinct proprietary approaches employed in managing DBA functionality.

Regarding DBA in FL, there are a few research approaches in PON enhancement to optimize bandwidth utilization. For instance, the authors of [7] proposed two techniques for DBA and wavelength allocation for PON (one based on statistical multiplexing for guaranteeing QoS for FL traffic across 50 Gbs Ethernet-PON and the second on bandwidth reservation). The aim in [7] was to let PON customers use their requested bandwidth for the FL application’s scheduling without compromising the QoS guarantees for other delay-critical applications. The suggested technique uses the well-known Differentiated Service approach to address the FL applications over the Ethernet PON QoS provisioning challenge. The bandwidth guarantee required for FL applications cannot be achieved simply by mapping FL traffic into a DiffServ per-hop behavior (PHB), as FL traffic would compete with traffic from other types of clients in the same PHB. It is possible to differentiate between FL traffic and other burst traffic by creating a PHB tailored for FL. This enables the DBA algorithm to prioritize the bandwidth according to the established policy.

The bandwidth-slicing method to support FL in edge computing is proposed in [6,12]. The proposed solution provides minimum communication delay for training traffic. According to the results, bandwidth slicing greatly increases training effectiveness while maintaining high learning accuracy. However, their approach failed to specify how to differentiate between FL traffic and other network traffic. The author of [13] proposed a solution of a client scheduling algorithm to balance the ONU load while securing the required bandwidth for FL traffic. However, their solution did not focus on providing advanced multiple queue management with prioritization to provide efficient bandwidth handling.

Furthermore, the authors of [14] proposed a DBA algorithm with an adaptive predictive model for low delay communications using the XGBoost [15] ensemble learning algorithm. Utilizing this technique, the authors claimed that the solution can maintain low delay communication characteristics despite environmental changes. The dynamic perception algorithm is inspired by reinforcement learning and adjusts according to environmental feedback. However, the adaptability and time-to-converge of this algorithm under various network scenarios have yet to be exhaustively analyzed. The paper does not explicitly mention the number of queues at the ONU or how the proposed algorithms interact with these queues. This can be a notable limitation.

In a TDM-PON, there are some novel DBA algorithms that are implemented to solve delay and bandwidth management. The authors of [16] proposed a learning-based solution named Online Convex Optimization (OCO) DBA. The main focus of [16] was to minimize the upstream delay by learning traffic delay over time. However, they utilized basic queue management. Immediate Allocation with Colorless Grant (IACG) DBA was introduced in [17] to decrease the delay of the fronthaul traffic. The solution efficiently allocates unallocated bandwidth to other ONUs. The Optimized Round-Robin (optimized-RR) was proposed in [18] to meet the restricted delay requirements of mobile fronthaul. The authors of [19] proposed the Efficient Bandwidth Utilization (EBU) DBA algorithm. The EBU allocates unused bandwidth to higher-demand ONUs. An extensive comparison of [16,17,18,19] is performed in Section 3.3 of this article.

Unlike existing bandwidth-based FL algorithms, our proposed model unlocks the power of PON’s control message to support the network and FL traffic separately. Furthermore, two problems are described: first, the FL model update aggregation at the ONU, and second, the DBA to support the system.

## 3. A Novel DBA-Based FL Aggregation over PON

**Figure 2:**
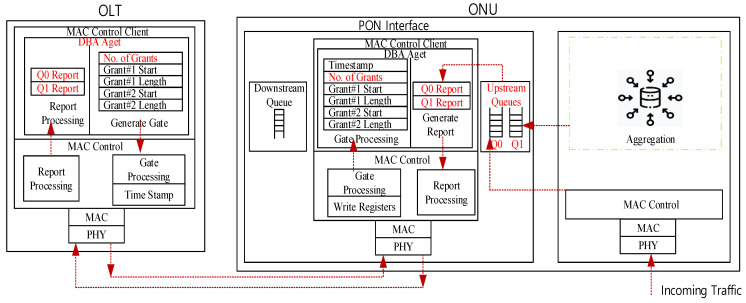
System overview.

This article assumes a Time-division Multiplexing (TDM)-PON system where an OLT is connected with multiple ONUs. Suppose that an ONU has two interfaces: one interface to the Customer Premises Equipment (CPE) (i.e., through a wireless antenna as a part of 6G) and a PON interface, as presented in Figure 2.

In Figure 2, we assume a two queue model: queue $Q_{0}$ is for network traffic (e.g., burst traffic), and queue $Q_{1}$ is for FL local model update traffic. When introducing FL traffic in PON, there can be two important optimizations required in the PON’s system described as follows:

- FL traffic aggregation at the ONU: Assuming that the ONU can classify the incoming upstream traffic from the CPEs, the traffic optimization problem is FL traffic aggregation. In FL, only model parameter updates are transmitted. Thus, the ONU needs to aggregate these updates from various devices in its domain before sending them upstream to the OLT and eventually to the central server. This can reduce the upstream traffic significantly.
- DBA optimization: As illustrated in Figure 2, assuming that the upstream queues at the ONU’s PON interface are dedicated to classified traffic ($Q_{0}$ for general traffic and $Q_{1}$ for FL aggregated model update), the role of the DBA at the OLT is to secure upstream bandwidth for these queues with proper fairness. Thus, the DBA should consider managing more than one queue bandwidth request. As defined in PON’s standards (e.g., [8,20]), the ONU sends a report control message to the OLT, reporting its upstream queue status. The OLT uses this information to grant an upstream bandwidth for the designated ONU. The report control message can carry information for different ONU’s queues, thanks to [8] standards. The DBA deals with reported queues separately, and issues two grants (one upstream bandwidth for each queue) in a single Gate control message (can carry multiple grants).

For more descriptions of the DBA optimization, the following subsection shows how we can deal with this problem.

### 3.1. Double-Queue DBA Algorithm for FL

In this subsection, we introduce a DBA algorithm that can manage the upstream bandwidth for all ONUs while each ONU has two upstream queues. The DBA solution in PONs must account for the number and nature of ONU queues (unlike in [14]). This requires the model to predict bandwidth grants for each queue separately. In this approach, the bandwidth requirements of each ONU are calculated based on their queue information (as reported from the ONU using a report control message). The OLT then allocates the available bandwidth to each ONU based on these calculations, intending to maximize the overall performance and efficiency of the FL system.

Suppose that there are *N* ONUs in the PON sharing bandwidth BW on each DBA cycle $T_{c}$; therefore, the total allocation decision on each $T_{c}$ is 2N. Assume that the bandwidth allocated to queue *j* of $ONU_{i}$ is defined as $b_{ij}$, where *j* is 0 or 1 and *i* is the ONU number (i∈N). For each queue *j* of $ONU_{i}$, suppose that the arrival rate is $\alpha_{ij}$ and each queue has a weight of $w_{ij}$; thus, the aim is to maximize the total efficiency for all ONUs based on a logarithmic function controlled by the arrival rate $\alpha_{ij}$, as presented in Equation (1), with the constraint in Equation (3).

(1) $$\begin{array}{cc} \mathrm{Maximize}\sum_{i=1}^{N} & \left((w_{i0}\cdot \log\left(\alpha_{i0}\cdot b_{i0}+1\right)\right)\\ \; & +(w_{i1}\cdot \log\left(\alpha_{i1}\cdot b_{i1}+1\right))), \end{array}$$

Therefore, the bandwidth allocated for $ONU_{i}$ must not surpass the maximum bandwidth, as in Equations (2) and (3).

(2) $$b_{i0}+b_{i1}\leq BW_{max,i}\;\mathrm{for}\;i\;=\;1,...,N.$$

(3) $$\sum_{i=1}^{N}\left(b_{i0}+b_{i1}\right)\leq BW$$

While logarithmic utility functions have been extensively adopted in wireless resource allocation problems due to their concave properties providing unique optima [21], they also bring inherent fairness into the allocation process, providing that resources are apportioned in a proportional-fair manner [22]. This fairness criterion is particularly important in PONs, where diverse services and applications coexist.

The optimization problem is a concave maximization problem with convex constraints (i.e., Equations (2) and (3) are linear). Solving Karush–Kuhn–Tucker (KKT) conditions can lead to finding the optimal BW allocation ($b_{ij}^{*}$). The Lagrangian for the optimization problem is formulated in Equation (4)

(4) $$\begin{array}{cc} L(b_{ij},\lambda ,\mu ,N,\alpha_{ij},w_{ij},BW,BW_{max})= & \sum_{i=1}^{N}(w_{i0}\log\left(\alpha_{i0}\cdot b_{i0}+1\right)\\ \; & +w_{i1}\log\left(\alpha_{i1}\cdot b_{i1}+1\right))\\ \; & -\lambda \left(\sum_{i=1}^{N}\left(b_{i0}+b_{i1}\right)-BW\right)\\ \; & -\sum_{i=1}^{N}\mu_{i}\left(b_{i0}+b_{i1}-BW_{max,i}\right). \end{array}$$

λ and $\mu_{i}$ in Equation (4) are Lagrange multipliers. We note here that the Lagrangian function for our optimization problem incorporates the objective function and the constraints with associated Lagrange multipliers. The dual problem is obtained by minimizing the Lagrangian with respect to the primal variables $b_{ij}$ while maximizing with respect to the dual variables λ and $\mu_{i}$. Table 1 presents the notations in this paper.

Algorithm 1 illustrates that the DBA is initialized to record the historical data ($b_{ij}\left(t-1\right)$, $b_{ij}\left(t-2\right)$, …) and the historical arrival rate ($\alpha_{ij}\left(t-1\right)$, $\alpha_{ij}\left(t-2\right)$, …).

**Figure 3:**
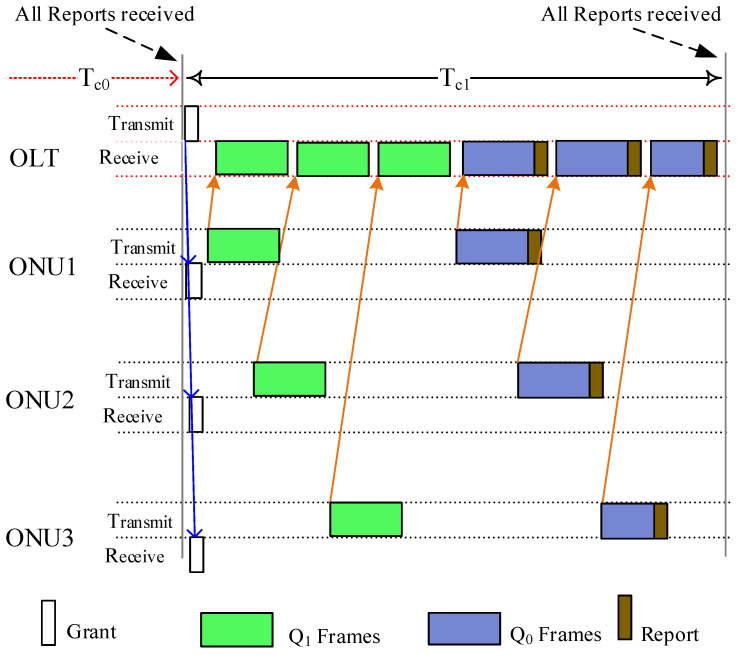
Bandwidth allocation on a DBA cycle $T_{c}$.

Figure 3 shows an example of how the bandwidth is managed on a DBA cycle. After the DBA cycle $T_{c0}$, all report control messages sent by the ONUs are received at the OLT. The OLT calculates the slot time for each queue of connected ONUs and sends the Gate control message to grant the bandwidth on $T_{c1}$. In this figure, we allocated two upstream grants (one for $Q_{0}$ and the second for $Q_{1}$ (for FL traffic)). The ONU uses the first grant to transmit FL-related frames from $Q_{1}$, as allocated at the beginning of the DBA cycle, and then uses the second grand to transmit the $Q_{0}$ frames and, afterward, sends the report control message reporting its current queues status at on $T_{c1}$. It has to be noted here that the narrow connection represents the frame transmission between the OLT and ONUs.
**Algorithm 1** Double-queue DBA algorithm for FL.  1:**Define the Lagrangian function:** Equation (4)  2:**Initialization:**  3:Initialize $b_{ij}$, λ, and  μ  4:Set initial values: for *N*, $\alpha_{ij}$, $w_{ij}$, BW, and $BW_{max}$  5:**Solve KKT conditions to find optimal bandwidth allocations:**  6:**while** not converged **do**  7:   **Update $b_{ij}$:**  8:   Calculate the Lagrangian gradient based on $b_{ij}$  9:   Update $b_{ij}$: $b_{ij}\leftarrow b_{ij}-\alpha \frac{\partial L}{\partial b_{ij}}$                                                // using gradient descent10:   Confirm that $b_{ij}$ is non-negative and within maximum bandwidth constraints11:   Update λ: $\lambda \leftarrow \lambda -\beta \left(\sum_{i=1}^{N}\left(b_{i0}+b_{i1}\right)-BW\right)$                    // based on Equation (3)12:   Update $\mu_{i}$: $\mu_{i}\leftarrow \max\left(0,\mu_{i}-\gamma \left(b_{i0}+b_{i1}-BW_{max,i}\right)\right)$         // based on Equation (2)13:**end while**14:**Allocate bandwidth to each ONU based on optimal solution:**15:**for** i=1 to *N* **do**16:   $ONU\left[i\right].queue\left[0\right].allocated\_bandwidth=b_{i0}$17:   $ONU\left[i\right].queue\left[1\right].allocated\_bandwidth=b_{i1}$18:**end for**19:**Convergence Check:**20:Check if the changes in $b_{ij}$, λ, and μ are below a predefined threshold21:If all changes are below the threshold, set converged to true

### 3.2. Simulation Model and Setup

**Figure 4:**
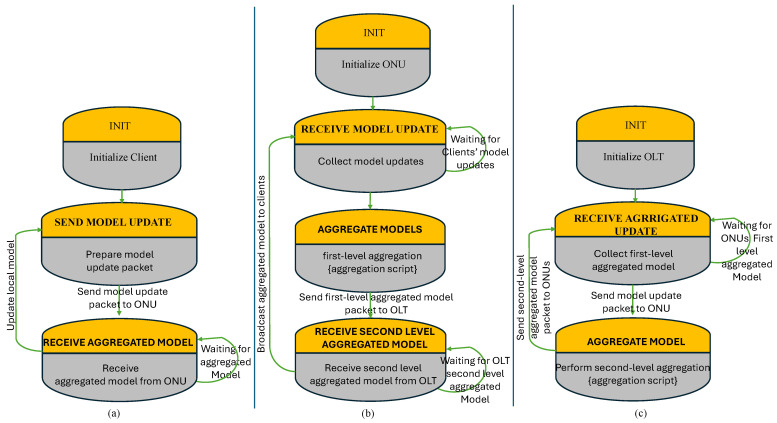
The process model of the (**a**) Client, (**b**) ONU, and (**c**) OLT.

**Figure 5:**
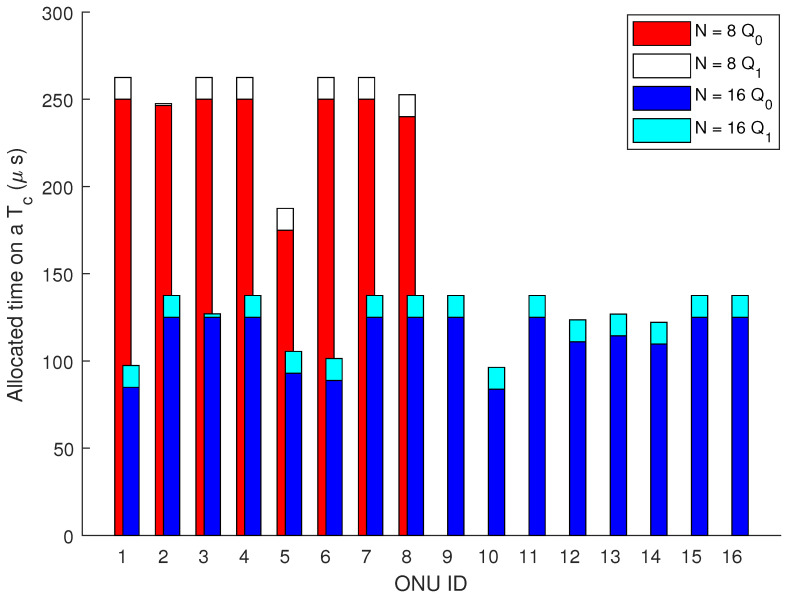
Bandwidth allocation example on a $T_{c}$ for 8 and 16 ONUs in PON.

We evaluated our proposed method for 8, 16, 32, and 64 ONUs. The data collection from the users has been performed using a uniform distribution method. We used our outstanding OPNET-PON model presented, for example, in [23,24]. The key challenge is bridging the gap between a tool that does not inherently support ML (OPNET) and a sophisticated ML-based prediction model. Therefore, we created a basic basic Python script for federated learning aggregation, and then simulate the communication process in OPNET. Thereby, to handle the communication stream and packet handling in OPNET, we utilized a ONPNET’s process model to provide that the transmission and reception of data representing the model updates and aggregated models and simulated correctly. Figure 4 represents the client process model (Figure 4a), the ONU process model (Figure 4b), and the OLT process model (Figure 4c). An example of bandwidth allocation of 8 and 16 ONUs scenarios is presented in Figure 5.

**Figure 6:**
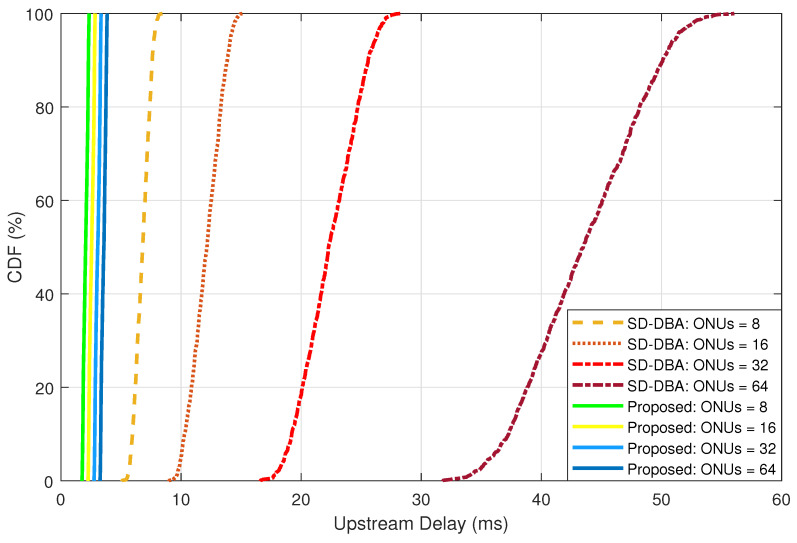
Evaluating the FL upstream delay.

We compared our proposed bandwidth allocation solution with the widely recognized SR-DBA, keeping the network conditions consistent. Both models were evaluated under the identical load of FL traffic emanating from 1000 IoT devices. The $T_{c}$ was set at 2 ms for both algorithms. As depicted in Figure 6, our proposed method consistently registered a delay ranging between 2 ms to 4 ms. In contrast, the SR-DBA algorithm showcased an escalating delay trend, particularly evident with a higher number of ONUs.

The reason behind this is that in our proposed solution, the upstream bandwidth stays the same regardless of how many FL IoT devices there are, given the use of the aggregation at the ONU. While the incoming traffic increases when the number of devices increases, the upstream traffic stays constant, similar to the bandwidth requirement of a single aggregated model. Our study shows the high efficiency of FL aggregation at the ONU level regardless of FL device count, as reported in [11]. On the other hand, stable bandwidth utilization provides network predictability and can reduce the upstream traffic delay. By giving an upstream grant for FL aggregated traffic on a $T_{c}$, the proposed solution shows that the proposed solution is faster than the existing solution.

The results indicate that our proposed DBA solution outperforms the existing SR-DBA method in terms of delay reduction, which is crucial for supporting real-time applications in 6G environments. Unlike the traditional SR-DBA, which struggles to maintain low delay with increasing ONUs, our model effectively manages the bandwidth by differentiating between FL and normal traffic and using multi-grants for improved efficiency.

**Figure 7:**
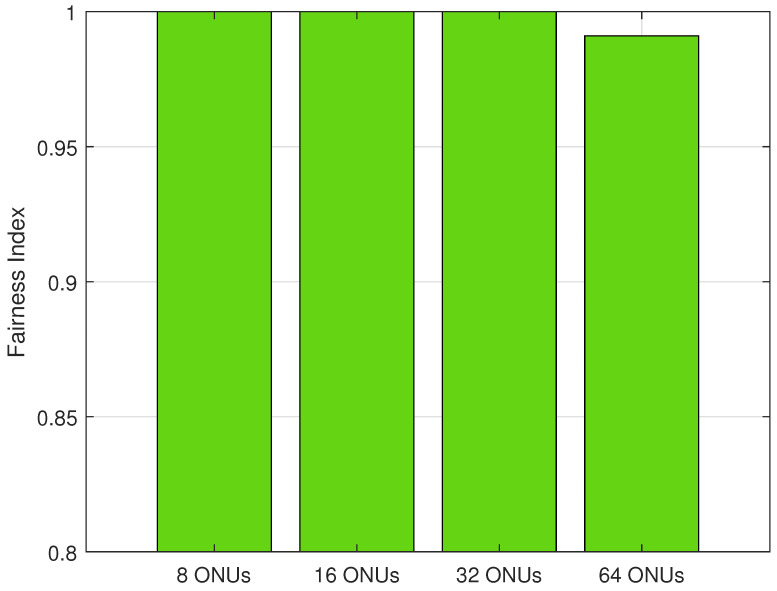
Fairness under different numbers on ONUs.

Figure 7 shows the fairness calculation of the proposed algorithm under different PON sizes (i.e., 8, 16, 32, and 64 ONUs). We utilize the results of Jain’s Fairness Index to measure how the bandwidth is fairly distributed for all ONUs. The result indicates that, when under 8 ONUs, 16 ONUs, and 32 ONUs, the proposed DBA algorithm is effectively fair in distributing the bandwidth for all the ONUs, whereas under the 64 ONUs scenario, the fairness is slightly decreased to 0.99 in the fairness index, which is not a significant reduction. This shows that, regardless of the number of ONUs in the PON, the proposed solution can maintain the bandwidth fairly for all ONUs. The logarithmic utility functions used in the proposed solution present inherent fairness into the allocation process and confirm that the bandwidth is allocated in a proportional-fair manner. This comparative analysis underscores the efficacy of our proposed model, particularly in terms of achieving lower delay and bandwidth fairness among the ONUs.

### 3.3. Comparison with Existing DBA Algorithms

Table 2 presents a comprehensive comparison of our proposed solution with some of the existing DBA solutions (i.e., [16,17,18,19]). The proposed DBA solution significantly reduces the upstream delay by optimizing the bandwidth allocation ONUs. This is achieved through the effective management of multiple queues at the ONU and prioritization of FL traffic. This method maximizes overall bandwidth utilization while maintaining fairness among ONUs. The proposed DBA algorithm solves a concave maximization problem with convex constraints, using a Lagrangian function to find the optimal bandwidth allocation for ONUs and ONUs’ queues, making sure that each queue is managed effectively.

## 4. Discussion and Open Challenges

### 4.1. Algorithm Complexity

As the number of ONUs increases, the time taken for bandwidth allocation or the data aggregation process might increase, potentially affecting the algorithm’s efficiency. Therefore, providing data privacy might introduce additional computational steps in an FL environment, adding to the complexity. Furthermore, for applications that require real-time communication, algorithms should not only be accurate, but also quick. DBA, especially in real-time scenarios, might pose challenges if the allocation algorithm does not keep up with the dynamic needs of the network. Managing multiple queues at the ONU and releasing multi-gate control messages can be complex, requiring sophisticated algorithms to handle them efficiently. Moreover, providing fair bandwidth distribution and maintaining QoS standards might introduce additional constraints to the algorithm, making it more complex. On the other hand, the parameters affecting the outcomes of the DBA algorithm include the arrival rates of traffic ($\alpha_{ij}$), the weights assigned to different queues ($w_{ij}$), and the total available bandwidth (BW). Further analysis can be employed to understand how these parameters influence the final bandwidth allocation decisions. For instance, we can analyze the impact of varying arrival rates on the allocation efficiency and delay to provide the robustness and fairness of the DBA solution.

The complexity of the proposed DBA algorithm is influenced by the number of ONUs and the number of queues managed by each ONU. The gradient descent method used for updating the bandwidth allocation in each iteration adds to the computational complexity. Additionally, solving the KKT conditions involves iterative updates of the Lagrange multipliers and dual variables, which increases the complexity further. Providing a balance between accuracy and computational efficiency is critical, especially as the number of ONUs increases. Future work should focus on optimizing the algorithm’s convergence time and computational overhead.

### 4.2. Coordination and Synchronization

In the FL-based PON system, the environment deals with a lot of CPE, which generates upstream traffic in PON. The control message technique builds the synchronization between CPE and ONU. The control message indicates which CPE is activated for the communication when CPE is connected to ONU. In other words, the control message builds the connection between CPE and ONU for better resource allocation. Strictly synchronizing time may not be necessary because each device performs the local training and global aggregation stages separately and independently. Each device has the ability to update its local model at its own pace and, when ready, interact with the aggregate server. Moreover, the aggregation windows method can determine which CPEs can send their local data to the ONU. Since precise strict synchronization is not required, this offers loose coordination and aids in managing the aggregation process.

### 4.3. Heterogeneity

In a TDM-PON, the downstream bandwidth is usually greater than the upstream bandwidth (the upstream is timely shared with all connected ONUs). However, the end devices must send their updated model parameters in FL, which the upstream bandwidth limitation can constrain. Moreover, these end devices might have different capabilities (e.g., computing power, memory, and bandwidth), which can delay the FL’s aggregation process.

### 4.4. Energy Efficiency in PON

Energy efficiency is an important part of PON standards. The energy saving is dedicated to the ONUs, and the energy-efficient techniques are defined for the fiber link interface. Three energy-saving modes are concluded in the latest ITU-T G-988 recommendation [20]: cyclic, Doze, and Watchful sleep modes.

- Cyclic sleep mode: The ONU transits its fiber links transmitter and receiver power mode between sleep and active cycles. In that case, the duration of each cycle is predefined (managed by the OLT), and could be configured based on the network requirements and the energy-saving algorithm.
- Doze mode: the transmitter components of the ONU are turned “OFF”, while the receiver is always “ON”.
- Watchful sleep mode: combines both the cyclic and doze modes by taking advantage of both modes and mitigating their drawbacks.

The energy-efficient mechanism in PON contributes to sustainable and environmentally friendly network operations, and on the other hand, it can provide efficient data communication. Selecting a reasonable energy-saving mode depends on the traffic pattern. For instance, the cyclic sleep mode can work perfectly in residential and business areas during the off-peak hours when there is not much traffic. Applying an energy-saving technique in PON under the FL scenario is an interesting topic. There are many challenges to address, such as developing a predictive energy-efficient mechanism, sleep and wake-up optimization, Coordination and Synchronization, and FL and energy efficiency, which are gaining researchers’ traction; therefore, research in this area can produce an outstanding modernization, optimizing resources and introducing PON collaborative learning.

## 5. Conclusions

In this paper, we have addressed an advanced intersection of FL from the 6G-powered PON framework. FL can be implemented in a large applicability domain given the high capabilities of 6Gs, such as ubiquitous connectivity. With regard to our work, it is apparent that a comprehensive DBA solution becomes important. Accordingly, this article focuses on two optimizations to embed FL traffic into a PON architecture: (1) the FL traffic aggregation at the ONU can reduce a large portion of upstream traffic, which possibly eliminates upstream PON traffic delay significantly; and (2) the DBA optimization must secure sufficient upstream bandwidth for the network and FL traffic, maintaining fairness between them. Results show that our proposed solution can provide significant improvement in delay and fairness compared to existing solutions. Moreover, we have discussed several challenges in the integration of the FL-based PON system. The availability of a large number of IoT devices and the ONUs need coordination and synchronization to provide better operations. The significance of Energy Efficiency in PON interestingly overlaps with multiple FL scenarios, revealing additional energy efficiency solutions creation prospects for PON. These challenges, together, shape the future research trajectory in the PON domain with the integration of FL in 6G.

## Figures and Tables

**Table 1:** Notation table.

Symbol	Definition
*N*	Number of ONUs in the PON
$T_{c}$	DBA cycle time
BW	Total available bandwidth
$BW_{max,i}$	Maximum bandwidth allocated to $ONU_{i}$
$b_{ij}$	Bandwidth allocated to queue *j* of $ONU_{i}$
$\alpha_{ij}$	Arrival rate for queue *j* of $ONU_{i}$
$w_{ij}$	Weight of queue *j* of $ONU_{i}$
λ	Lagrange multiplier for total bandwidth constraint
$\mu_{i}$	Lagrange multiplier for maximum bandwidth constraint for $ONU_{i}$
*L*	Lagrangian function
$Q_{0}$	Queue for network traffic
$Q_{1}$	Queue for FL local model update traffic

**Table 2:** Comparison with different DBA algorithms.

Item	Proposed DBA	Learning Based DBA [16]	IACG DBA [17]	Optimized-RR DBA [18]	EBU DBA Algorithm [19]
**DBA Algorithm Approach**	Double Queue Management	Online Convex Optimization (OCO) DBA	Immediate Allocation with Colorless Grant (IACG) DBA	Optimized Round-Robin (optimized-RR)	Efficient Bandwidth Utilization (EBU) DBA algorithm
**Delay**	Reduce delay significantly by optimizing bandwidth for multiple queues at the ONU efficiently and prioritizing FL Traffic	Low average delay	Achieves 99.96% of fronthaul delay requirements at 80% load	Achieves mobile fronthaul delay requirements of 300 μs	Reduces mean delay compared to older solutions
**Bandwidth Utilization**	Provides high efficiency to satisfy FL traffic demands	Slice prioritization weight is used to tune delay sensitive ONUs	Efficient allocation to satisfy xHaul transport network requirements	Improved utilization for mobile fronthaul	Utilizes unused bandwidth
**ONU Queue Management**	Advanced (Multiple Queues, one of them for traffic FL)	Basic Queue Management	Intermediate Queue Management	dynamic maximum allocation	Polling scheme
**Fairness**	Fairness with minimum allocation for all ONUs	Fairness with delay, however; allocation is based on slice prioritization weight metrics	Equally Allocates with colorless grant	Fair allocation with dynamic maximum allocation	Allocates unused bandwidth to ONUs with higher demand

## Data Availability

Data are contained within the article.

## Abbreviations

The following abbreviations are used in this manuscript:

5G	Fifth Generation
6G	Sixth Generation
AI	Artificial Intelligence
Alloc-ID	Allocation Identifier
CPE	Customer Premises Equipment
DBA	Dynamic Bandwidth Allocation
DWBA	Dynamic Wavelength and Bandwidth Allocation
E-PON	Ethernet Passive Optical Network
FL	Federated Learning
G-PON	Gigabit Passive Optical Network
IoT	Internet of Things
KKT	Karush–Kuhn–Tucker
ML	Machine Learning
ONU	Optical Network Unit
OLT	Optical Line Terminal
PON	Passive Optical Network
QoS	Quality of Service
SR-DBA	Status Reporting Dynamic Bandwidth Allocation
TDM-PON	Time-Division Multiplexing Passive Optical Network
TM-DBA	Traffic Monitoring Dynamic Bandwidth Allocation
VR	Virtual Reality
XGS-PON	10-Gigabit-Capable Symmetric Passive Optical Network
